# Impacts of Physical Inactivity Models on Endothelial Function: A Systematic Review

**DOI:** 10.1007/s40279-025-02238-x

**Published:** 2025-05-19

**Authors:** Joel E. Harden, J. David Branch, Leryn J. Reynolds

**Affiliations:** 1 Integrative Cardiometabolic Research Laboratory, School of Exercise Science, Old Dominion University, 1006C Student Recreation CTR, Norfolk, VA 23529 USA; 2 School of Exercise Science, Old Dominion University, Norfolk, VA USA

## Abstract

**Background:**

Endothelial dysfunction is associated with cardiovascular disease and cardiac events. Numerous studies demonstrate that a reduction in physical activity/exercise levels are associated with poor endothelial function. Yet, these studies use a plethora of models to mimic reduced activity levels which may have widely different results on endothelial function. It is pertinent to collectively review these articles to provide a comprehensive understanding of the impact of reduced activity on vascular health, as endothelial function is one of many factors that influences vascular tone.

**Objective:**

The purpose of this systematic review is to examine and synthesize the current literature regarding the effects of acutely (≤ ~ 2 months) reducing physical activity on endothelial function.

**Methods:**

This systematic review contains a search of two databases (PubMed, Web of Science) conducted by two reviewers. After screening and review, the search yielded 20 studies that were appraised and reviewed.

**Results:**

Articles were separated into four categories based on the type of inactivity intervention: reduction in daily physical activity interventions, detraining (removal of exercise) interventions, bed rest interventions, and immobilization interventions. Acute physical inactivity interventions were largely shown to reduce upper and lower limb artery flow-mediated dilation and lower limb microvascular function.

**Conclusion:**

The results indicate that those studies with increased time spent in inactivity, as well as increased severity of inactivity, were more likely to have negative endothelial function outcomes. Future research should examine differences in the severity of physical inactivity interventions regarding endothelial function.

## Key Points

**Table:** 

Interventions enforcing removal of physical activity or exercise tend to impair endothelial function.
Studies with increased time and severity of inactivity were more likely to have negative endothelial outcomes.

## Introduction

Human blood vessels are lined with endothelial cells, which have an important role in the dilation and constriction of blood vessels, also known as vascular tone [1]. The primary way in which endothelial cells regulate vascular tone is through secretion of factors that stimulate vasodilation or vasoconstriction of blood vessels [1]. One of the most common ways that macrovascular endothelial function is measured is via the flow-mediated dilation (FMD) technique [2]. FMD is a noninvasive technique of assessing endothelial function using high-resolution Doppler ultrasound to image the change in diameter of an artery from pre to post reactive hyperemia [3]. This technique is widely recognized as a valid and reliable measure of macrovascular endothelial function [2, 4], which is commonly expressed as a percent change between baseline diameter and peak diameter following reactive hyperemia (%FMD). Microvascular endothelial function can be assessed via laser Doppler flowmetry, a technique that uses laser Doppler to measure human skin blood flow, usually in response to the infusion of acetylcholine [5]. Impaired endothelial function is thought to be the first marker of atherosclerosis and is thus considered a marker for cardiovascular disease (CVD) [6]. CVD is the number one cause of death in the United States of America (USA) [7] and globally [8] and accounted for an estimated US $400 billion in 2020 in healthcare costs in the USA [9]. While many factors can impact endothelial function, one modifiable lifestyle factor that may contribute to impaired endothelial function is physical inactivity.

Approximately 25% of adults in the USA are physically inactive [10]. In fact, physical inactivity has been termed as the “biggest public health problem of the twenty-first century” [11]. Among a plethora of diseases, physical inactivity is linked to cardiovascular disease (CVD) [12], of which a primary indicator is endothelial dysfunction. Additionally, physical inactivity can lead to microvascular dysfunction [13], which can contribute to CVD [14, 15]. Reducing daily physical activity may lead to a reduction in endothelial function [16–18]. O’Brien et al. [19] found that older individuals who did not meet adequate levels of moderate to vigorous physical activity (MVPA), compared with those who did meet adequate levels of MVPA, had lower brachial and popliteal artery %FMD. Numerous studies [20–25], which we review in this systematic review, have demonstrated that reducing activity levels results in lowered %FMD. These studies utilize different models to induce inactivity and examine the impact of inactivity on both macrovascular and microvascular endothelial function.

The studies examining reducing daily physical activity range in severity and model of reduced daily activity. Four such models are discussed in this review. The first model examines a reduction of free-living daily physical activity by reducing total daily steps taken below the level deemed “active” (10,000) [26]. The second model examines detraining and involves the cessation of exercise in individuals previously exercising. The third model examines total or near-total removal of physical activity and is implemented either as bed rest or as dry immersion (placing a subject in a tank of water while keeping them dry, such as by wearing a waterproof suit, to simulate weightlessness). Finally, the last model examines immobilization, usually of a single limb, through the use of a sling, cast, or other device capable of rendering a portion of the body inert for a period of time. While each of these interventions induces a reduction in activity, both the severity and the methodology of the interventions differ, and thus merit individual consideration.

Despite this plethora of evidence regarding physical activity and endothelial function [16, 18, 27–32], no reviews have been published, to the authors’ knowledge, regarding the effects of short-term inactivity on endothelial function among healthy adults. The results of a systematic review of this nature may be important in understanding how quickly changes in endothelial function occur. Understanding the speed and process by which changes in endothelial function occur is key to our understanding of the pathogenesis of cardiovascular disease. The aim of this systematic review is to evaluate the impact of short-term physical inactivity and reduction of exercise on measures of endothelial function in adults.

## Methods

### Design

This review is reported in accordance with the Preferred Reporting Items for Systematic Reviews and Meta-Analyses (PRISMA) [33] guidelines, and utilized the Population, Intervention, Comparison, and Outcomes (PICO) [34] question, “What is the effect of short-term physical inactivity on macrovascular and microvascular endothelial function of adults without known cardiovascular disease?” Electronic database searches were performed in two databases (PubMed and Web of Science) from earliest record to October 2024. The search strategy, presented in Table 1, combined “adult” with a variety of terms/phrases representing imposed physical inactivity interventions and a variety of terms/phrases describing endothelial health outcomes.

**Table 1:** Overview of the search strategy

Variable	Search terms
Group 1: population terms	Adult
Group 2: inactivity terms	“Reduced activity” OR “physical inactivity” OR “acute inactivity” OR “inactivity” OR “reduced exercise” OR “short-term physical inactivity” OR “short-term inactivity” OR “decreased physical activity” OR “decreased activity” OR “detraining” OR “short-term detraining” OR “deconditioning” OR “immobilization” OR “bed rest”
Group 3: outcome terms	“Flow-mediated dilation” OR “flow mediated dilation” OR “endothelial function” OR “endothelial dysfunction” OR “endothelial dependent vasodilation” OR “endothelin-1” OR “nitric oxide” OR “microvasculature” OR “microcirculation” OR “contrast-enhanced ultrasound” OR “near-infrared spectroscopy” OR “laser doppler flowmetry” OR “microvascular function” OR “thermodilution” OR “capillary microscopy” OR “retinal imaging”

No filters or limits were used in the search strategy

### Inclusion and Exclusion Criteria

Studies were included if the physical inactivity intervention consisted of removal of structured exercise from otherwise healthy individuals, and/or included reduction in daily physical activity levels, for at least 3 days and no longer than 60 days. Interventions were only included if pre-intervention testing was done during a state in which exercise and physical activity was allowed. Only experimental interventions were included. Also, only studies examining a measure of macrovascular (%FMD) or microvascular (laser Doppler) endothelial function were eligible. Studies examining all forms of reduction of exercise and physical activity (including step reduction, removal of exercise, dry immersion, limb immobilization or suspension, and bed rest) were considered for inclusion.

### Participants

Trials that were completed with adults (age 18 + years) who were healthy and had no known endothelial disorders at the time of inclusion in the study were included.

### Outcome Measures

Eligible trials reported measures of macrovascular endothelial function via flow-mediated dilation or of microvascular function via laser Doppler flowmetry, before and after a physical inactivity intervention in active adults.

### Selection of Studies

All records of studies meeting search criteria were downloaded from PubMed and Web of Science and formatted into a Microsoft Excel document. Duplications were eliminated, and two investigators independently screened the results against the eligibility criteria. Articles were first screened through title and abstract within the Excel document. If it was still unclear whether an article met the inclusion criteria, the full text was read. A consensus was then reached by discussion, and all disagreements were resolved by a discussion. The selected articles were evaluated in their entirety, with all relevant information such as participant characteristics, sample sizes, study designs, and outcome measures extracted.

### Study Quality

Studies were assessed for risk of bias (“low”, “high”, “some concerns”) via the Version 2 Cochrane Risk of Bias Tool for Assessing Risk of Bias in Randomized Trials [35] for all included randomized trials. All observational cohort studies were assessed for study quality (“good”, “fair”, “poor”) via the National Heart, Lung, and Blood Institute (NHLBI) Quality Assessment Tool for Observational Cohort and Cross-Sectional Studies [36]. Study risk of bias outcomes are reported in Tables 3, 4.

### Synthesis and Presentation of Results

Studies were grouped and examined on the basis of on their interventions. Subcategories based on interventions were as follows: reduction in daily physical activity, detraining, bed rest, immobilization. Study outcomes are reported in Table 2.

**Table 2:** Study characteristics

Study	Study design/participants	Intervention	Outcome measures	Results	Model
Bowden-Davies et al., 2021 [20]	Single cohort pre-test post-test *N* = 28 (age 32 ± 11 years)	Subjects completed 14 days of daily step reduction (< 1500 steps per day) followed by 14 days of return to habitual step daily levels	Brachial artery FMD	%FMD decreased from baseline [8.1 (6.8, 9.3)] to 14 days of step reduction [6.2 (5.2, 7.3)]. 14 days of return to habitual daily steps levels increased %FMD [7.6 (6.5, 8.8)] back to baseline. Data are presented as mean (confidence interval)	Reduction in daily steps
Boyle et al., 2013 [21]	Single cohort pre-test post-test *N* = 11 (25 ± 2 years)	Subjects completed 5 days of reduced daily physical activity (cessation of exercise, reduced daily steps from > 10,000 to < 5000) in free-living conditions. Assessments were taken at baseline and on days 3 and 5 of physical inactivity	Popliteal and brachial artery FMD	2.98% decrease in popliteal artery %FMD. Brachial artery %FMD did not change. 0.23 mm decrease in diameter of brachial artery but not popliteal artery	Reduction in daily steps
Teixeira et al., 2017 [22]	Single cohort pre-test post-test *N* = 13 (20 ± 2 years)	All participants underwent 5 days of reduced physical activity and reduced steps (cessation of exercise, reduced daily steps from > 10,000 to < 5000). One leg was submerged in warm water three times per day for 30 min each during the physical inactivity intervention while the contralateral leg was not	Popliteal artery FMD	%FMD decreased from baseline [6.82 ± 0.30] to 5 days of step reduction [6.47 ± 0.36]. Data are presented as mean ± SE	Reduction in daily steps
Gill et al., 2003 [39]	Single cohort pre-test post-test *N* = 8 (27.8 ± 12.1 years)	Subjects completed one week of regular exercise training followed by 1 week of no exercise training. Assessments were performed during both weeks	Laser Doppler analysis of microvascular response to acetylcholine	17% decrease and 22% decrease in microvascular function in the trained and detrained state, respectively. No differences in the percentage of decrease between states was found	Detraining
Hunt et al., 2012 [40]	Single cohort pre-test post-test *N* = 9 (age 26 ± 4 years)	Subjects completed 4 weeks of dynamic handgrip training, with one forearm trained with blood flow restriction while the other was trained without blood flow restriction. Following training, subjects underwent 2 weeks of detraining. Measurements were taken pre-training, post-training, and post-detraining	Brachial artery FMD	Brachial artery %FMD showed no differences in either arm between pretraining [BFR arm: 6.5 ± 2.7; control arm: 6.9 ± 1.6], post training [BFR arm: 5.7 ± 2.0; control arm: 6.6 ± 2.6], and detraining [BFR arm: 6.7 ± 2.4; control arm: 6.3 ± 2.5]. Data are presented as mean ± SE	Detraining
Wang, 2005 [41]	Single cohort pre-test post-test *N* = 10 (21.6 ± 0.2 years)	Subjects underwent 8 weeks of exercise training followed by 8 weeks of cessation of training. Subjects were measured prior to and following training, and after detraining	Laser Doppler analysis of microvascular response to acetylcholine	Skin blood flow in the forearm increased from pretraining [178.3 ± 12.7, mL/min] following exercise [414.2 ± 57.1, mL/min]; these levels returned to baseline following detraining [177.8 ± 25.1, mL/min]. Data are presented as mean ± SE	Detraining
Bleeker et al., 2005 [48]	RCT with small sample size *N* = 16 (34 ± 2 years)	Subjects were randomly separated into two groups, which underwent either bed rest only or bed rest with resistive vibration exercise. All subjects underwent 52 days of bed rest, with measurements taken prior to bed rest, following 25 days, and following 52 days	Femoral artery FMD	17% decrease in femoral artery diameter and 4% increase in femoral artery %FMD in the control group only	Bed rest
Demiot et al., 2007 [38]	RCT with small sample size *N* = 16 (33.5 ± 0.9 years)	Both groups underwent 20 days of ambulation, 60 days of bed rest, and another 20 days of ambulation. The control group completed no activity during bed rest, while the active group completed a laying-down exercise protocol consisting of both resistance and aerobic exercise for 30 min each at a time	Laser Doppler analysis of microvascular response to acetylcholine	Lower body endothelium-dependent vasodilation decreased from baseline [35.4 ± 4.8, %] following bedrest [24.1 ± 3.8, %] in the control group. Data are presented as mean ± SE	Bed rest
Dyson et al., 2007 [23]	RCT with small sample size *N* = 24 (33.5 ± 0.9 years)	Subjects underwent 20 days of ambulation, 60 days of bed rest, and another 20 days of ambulation. The control group completed no activity during the bed rest, while the active group completed a laying-down exercise protocol consisting of both resistance and aerobic exercise for 30 min each at a time	Brachial and popliteal artery FMD	No differences were observed regarding %FMD	Bed rest
Hamburg et al., 2007 [13]	Single cohort pre-test post-test N = 20 (30.7 ± 8 years)	Subjects underwent 5 days of bed rest for 23.5 h per day. Measurements were taken at baseline and following 5 days of bed rest	Brachial artery FMD and reactive hyperemia	Reactive hyperemia was found to decrease in the forearm from baseline [1317 ± 404, mL/min] following bed rest [1112 ± 260, mL/min]. Reactive hypermia decreased at the calf from baseline [28.5 ± 7.0, mL/min] following bed rest [22.2 ± 8.7, mL/min]. No change in brachial artery %FMD was observed. Data are presented as mean ± SE	Bed rest
Navasiolava et al., 2010 [43] and 2011 [42]	Single cohort pre-test post-test *N* = 8 (23 ± 0.5 years)	Participants underwent 7 days of dry immersion, with complete or near-complete physical inactivity	Laser Doppler analysis of microvascular response to acetylcholine	Reduced basal flow and calf endothelium-dependent vasodilation from baseline [22 ± 4 a.u.] to 7 days of dry immersion [11 ± 1 a.u.)]. Increase in circulating endothelial microparticles (increase of 23 EMPs/microl) on day 3. Data are presented as mean ± SE	Bed rest
Nosova et al., 2014 [24]	Single cohort pre-test post-test *N* = 5 (22 ± 2 years)	Participants underwent 5 days of bed rest, with complete or near-complete physical inactivity	Brachial and femoral artery FMD	%FMD decreased from baseline [11 ± 3] following bed rest [9 ± 2] in the brachial artery. %FMD also decreased from baseline [4 ± 1] following bed rest [2 ± 1] in the femoral artery. Data are presented as mean ± SE	Bed rest
Platts et al., 2009 [25]	Single cohort pre-test post-test *N* = 13	Subjects underwent 49–60 days of bed rest. Measurements were taken at baseline and following 7, 49, and 60 days of bed rest	Popliteal artery FMD	6% increase from baseline to 49 days of bed rest in popliteal artery %FMD	Bed rest
Yuan et al., 2015 [49]	RCT with small sample size *N* = 14 (33.5 ± 0.9 years)	Subjects underwent 60 days of head down tilt bed rest. Subjects were split into a supplement group that was given an herb (Taikong Yangxin), and a control group that was not given this herb. Measurements were taken 15 days prior to bed rest and following bed rest	Laser Doppler analysis of microvascular response to acetylcholine	30% decrease in calf endothelium-dependent vasodilation following bed rest in the control group	Bed rest
Birk et al., 2012 [44]	Single cohort pre-test post-test *N* = 13 (22 ± 1 years)	Subjects underwent 8 days of forearm immobility wearing a sling over their non-dominant arm. Measures were taken prior to, following 4 days, and following 8 days of immobilization	Brachial artery FMD	No change in %FMD was found, but a significant decrease in peak blood flow was found in the immobilized arm after 8 days	Immobilization
Bleeker et al., 2005 [45]	Single cohort pre-test post-test *N* = 7 (24 ± 2 years)	Subjects underwent 4 weeks of unilateral lower limb suspension. Measurements were taken prior to and following the 4 week intervention	Femoral artery FMD	Calf blood flow decreased at baseline [2.1 ± 0.2, mL/min/dL] following suspension [1.6 ± 0.2, mL/min/dL]. No change in femoral artery %FMD was found. Data are presented as mean ± SE	Immobilization
Cohen et al., 2021 [37]	RCT with small sample size *N* = 31 (22.4 ± 3.7 years)	31 subjects were split into three groups: a control group that underwent only single-leg immobilization; a BFR group that underwent immobilization and used blood flow restriction training; and a group that underwent immobilization and utilized blood flow restriction and electric muscle stimulation (EMS). Immobilization lasted for 14 days, and measurements were taken prior to and following immobilization	Femoral artery FMD	%FMD was unchanged between groups at baseline [control: 5.12 ± 3.8, BFR: 7.22 ± 3.4, BFR + EMS: 4.80 ± 1.4] and following immobilization [control: 5.00 ± 3.6, BFR: 8.33 ± 3.7, BFR + EMS: 6.83 ± 4.4]. Data are presented as mean ± SE	Immobilization
Rakobowchuk et al., 2011 [46]	Single cohort pre-test post-test *N* = 15 (20.8 ± 0.7 years; 20.4 ± 0.8 years)	Subjects underwent 12 days of single leg immobilization. Measures were taken prior to and following immobilization	Popliteal artery FMD	%FMD increased in the popliteal artery from baseline [6.4 ± 1.3] following immobilization [13.5 ± 2.7]. Data are presented as mean ± SE	Immobilization
Rytter et al., 2020 [47]	Single cohort pre-test post-test *N* = 12 (22.1 ± 0.4 years)	Subjects underwent 14 days of single leg immobilization. Measures were taken prior to and following immobilization	Laser Doppler analysis of microvascular response to acetylcholine	30% decrease in vascular conductance following immobilization	Immobilization

*FMD* flow-mediated dilation, *NHLBI* National Heart, Lung, and Blood Institute, *RCT* randomized controlled trial, *BFR* blood flow restriction, *EMP* endothelial microparticles, *SE* standard error, *EMS* electric muscle stimulation, *a.u.* arbitrary units

## Results

### Study Characteristics

**Fig. 1:**
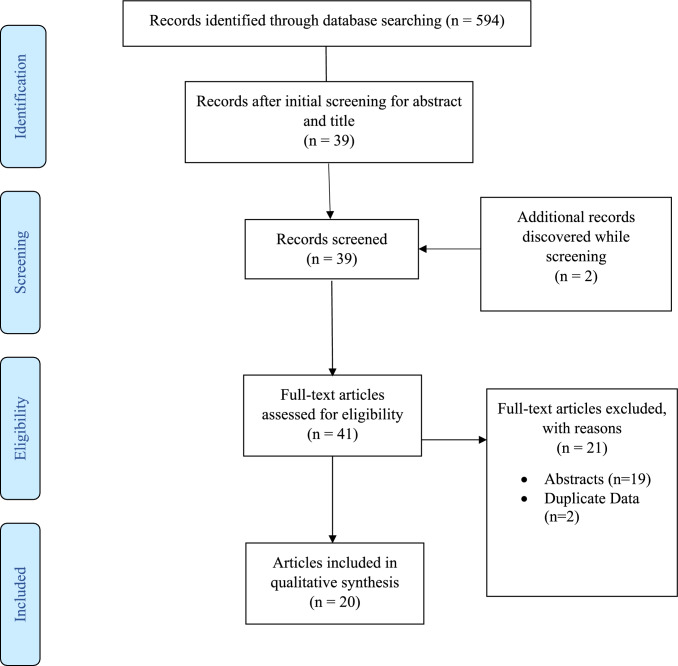
PRISMA search strategy. An overview of the search strategy as stated in PRISMA guidelines [33], from identification of potential studies to all included studies

The original search yielded a total of 594 records (398 from PubMed and 196 from Web of Science). Of these, 555 were eliminated because their abstracts or titles indicated that they did not meet the inclusion criteria. The full text of the remaining 39 articles was read, and 21 were eliminated on the basis of inclusion criteria or because of duplicate data, leaving 18 records remaining. The references of included articles were reviewed, and 2 additional articles were included, leaving 20 studies remaining for full analysis. Sample sizes ranged from 5 [24] to 31 [37] participants, with a total of 273 participants summed across all studies, while sex participation ranged from 0% [21] to 100% female [23, 38]. All 20 of the studies were experimental trials, with 15 [13, 20–22, 24, 25, 39–47] single-cohort pre-test post-test studies and 5 [23, 37, 38, 48, 49] randomized controlled trials with small sample sizes (Table 2). Mean ages ranged from 20 ± 2 years [22] to 34 ± 2 years [48]. The search/selection process is shown in Fig. 1, and study characteristics are presented in Table 2.

### Description and Outcomes of Studies

Each of the 20 studies reduced daily physical activity or exercise levels in their participants. However, the physical inactivity interventions differed between studies in both length of time and type of inactivity. Study description and outcomes are presented in Table 2. Detailed results from the Cochrane Risk of Bias Assessment and the NHLBI Quality Assessment Tool for Observational Cohort and Cross-Sectional Studies are presented in Tables 3, 4.

**Table 3:** National Heart, Lung, and Blood Institute quality assessment tool results

Study	Q1	Q2	Q3	Q4	Q5	Q6	Q7	Q8	Q9	Q10	Q11	Q12	Q13	Q14	Overall
Bowden-Davies et al., 2021 [20]	Yes	Yes	Yes	Yes	Yes	Yes	Yes	No	Yes	No	Yes	No	Yes	Yes	Fair
Boyle et al., 2013 [21]	Yes	Yes	Yes	Yes	No	Yes	Yes	No	Yes	No	Yes	No	Yes	Yes	Fair
Teixeira et al., 2017 [22]	Yes	Yes	Yes	No	No	Yes	Yes	No	Yes	No	Yes	No	Yes	No	Fair
Gill et al., 2003 [39]	Yes	Yes	Yes	Yes	No	Yes	Yes	No	Yes	No	Yes	No	Yes	Yes	Fair
Hunt et al., 2012 [40]	Yes	Yes	Yes	Yes	No	Yes	Yes	No	Yes	No	Yes	No	Yes	Yes	Fair
Wang, 2005 [41]	Yes	Yes	Yes	No	No	Yes	Yes	No	Yes	No	Yes	No	Yes	No	Fair
Hamburg et al., 2007 [13]	Yes	Yes	Yes	Yes	Yes	Yes	Yes	No	Yes	No	Yes	Yes	Yes	Yes	Good
Navasiolava et al., 2010 [43] and 2011 [42]	Yes	Yes	Yes	Yes	No	Yes	Yes	No	Yes	No	Yes	No	Yes	Yes	Fair
Nosova et al., 2014 [24]	Yes	Yes	Yes	Yes	No	Yes	Yes	No	Yes	No	Yes	No	Yes	Yes	Fair
Platts et al., 2009 [25]	Yes	Yes	Yes	Yes	No	Yes	Yes	No	Yes	No	Yes	No	Yes	Yes	Fair
Birk et al., 2012 [44]	Yes	Yes	Yes	Yes	No	Yes	Yes	No	Yes	No	Yes	No	Yes	Yes	Fair
Bleeker et al., 2005 [45]	Yes	Yes	Yes	Yes	No	Yes	Yes	No	Yes	No	Yes	No	Yes	Yes	Fair
Rakobowchuk et al., 2011 [46]	Yes	Yes	Yes	Yes	No	Yes	Yes	No	Yes	No	Yes	No	Yes	Yes	Fair
Rytter et al., 2020 [47]	Yes	Yes	Yes	Yes	Yes	Yes	Yes	No	Yes	No	Yes	No	Yes	Yes	Fair

Assessment of cohort study quality based on 14 criteria from the National Heart, Lung, and Blood Institute (NHLBI) Quality Assessment tool criteria are as follows:

Q1. Was the research question or objective in this paper clearly stated?

Q2. Was the study population clearly specified and defined?

Q3. Was the participation rate of eligible persons at least 50%?

Q4. Were all the subjects selected or recruited from the same or similar populations (including the same time period)? Were inclusion and exclusion criteria for being in the study prespecified and applied uniformly to all participants?

Q5. Was a sample size justification, power description, or variance and effect estimates provided?

Q6. For the analyses in this paper, were the exposure(s) of interest measured prior to the outcome(s) being measured?

Q7. Was the timeframe sufficient so that one could reasonably expect to see an association between exposure and outcome if it existed?

Q8. For exposures that can vary in amount or level, did the study examine different levels of the exposure as related to the outcome (e.g., categories of exposure, or exposure measured as continuous variable)?

Q9. Were the exposure measures (independent variables) clearly defined, valid, reliable, and implemented consistently across all study participants?

Q10. Was the exposure(s) assessed more than once over time?

Q11. Were the outcome measures (dependent variables) clearly defined, valid, reliable, and implemented consistently across all study participants?

Q12. Were the outcome assessors blinded to the exposure status of participants?

Q13. Was loss to follow-up after baseline 20% or less?

Q14. Were key potential confounding variables measured and adjusted statistically for their impact on the relationship between exposure(s) and outcome(s)?

**Table 4:** Cochrane risk of bias tool results

Study	Domain 1 (low, high, some concerns)	2	3	4	5	Overall (low, high, some concerns)
Bleeker et al., 2005 [48]	Low	Low	Low	Low	Low	Low
Demiot et al., 2007 [38]	Low	Low	Low	Low	Low	Low
Dyson et al., 2007 [23]	Low	Low	Low	Some Concerns	Low	Low
Yuan et al., 2015 [49]	Low	Low	Low	Low	Low	Low
Cohen et al., 2021 [37]	Low	Low	Low	Low	Some Concerns	Low

Assessment of randomized controlled trial study risk of bias based on five domains from the Cochrane Risk of Bias Tool:

Domain 1: Risk of bias arising from the randomization process

Domain 2: Risk of bias due to deviations from the intended interventions

Domain 3: Missing outcome data

Domain 4: Risk of bias in measurement of the outcome

Domain 5: Risk of bias in selection of the reported result

#### Reductions in Daily Steps

In the three studies examining reductions in daily physical activity levels, inactivity interventions ranged from 5 days [21, 22] to 14 days [20]. Reductions in daily step counts were used by Boyle et al. [21] (reduce daily steps from > 10,000 to < 5000 for 5 days), Bowden-Davies et al. [20] (reduce step count to < 1500 steps per day for 14 days), and Teixera et al. [22] (reduction of steps from ≥ 10,000 per day to ≤ 5000 steps per day for 5 days).

All of the above studies examined %FMD. Both Teixera et al. [22] and Boyle et al. [21] found significantly lower popliteal %FMD following their 5-day inactivity interventions, while neither found a significant decrease in brachial %FMD. Boyle et al. [21] observed a %FMD reduction of 2.98% in the popliteal artery, while Teixera et al. [22] observed a %FMD reduction of 3.76% in the popliteal artery. Bowden-Davies et al. [20] found a significant decrease in brachial artery %FMD by 1.8% following a 14-day reduced step count intervention, but did not measure popliteal %FMD. Following a return to habitual physical activity levels, %FMD increased by 1.4%, which was no longer statistically significantly different from baseline %FMD.

#### Detraining

In the three studies examining detraining, physical inactivity interventions ranged from 1 week [39] to 8 weeks [41]. Of the three studies, all employed an exercise intervention in individuals who habitually did not exercise, followed by a period of detraining [39–41]. Hunt et al. [40] found no difference in brachial artery %FMD following detraining. Gill et al. [23] examined the vasodilator response to the iontophoresis of acetylcholine using laser Doppler imagery 4 h postprandially and found significant decreases in both a trained (reduction of 17%) or detrained (reduction of 22%) state after 1 week of detraining. No differences were observed between the degree of reduction in the trained and detrained states. In contrast, Wang et al. [41] found a significant decrease in microvascular function following 8 weeks of detraining that was not observed in a trained state.

#### Bed Rest

In the studies examining bed rest (seven studies) or dry immersion (two studies) interventions, the interventions ranged from 5 days [13, 24, 42, 43] to 60 days [23, 25, 38, 49]. Two of the nine studies had participants undergo 20 days of ambulation followed by 60 days of bed rest, and another 20 days of ambulation [23, 38]. Four studies had subjects undergo 5 days [13, 24] or 60 days [25, 49] of bed rest, with study measures taken prior to and following the bed rest intervention. The remaining bed rest study had participants separated into two groups, one of which underwent bed rest while the other underwent bed rest with resistive vibration exercise [48]. Lastly, while not considered bed rest, two studies from the same sample had participants undergo 7 days of dry immersion, which induces complete or near-complete physical inactivity similar to bed rest [42, 43].

Five of the nine bed rest studies examined %FMD. Bleeker et al. [32] found a significant increase of 4% in femoral artery %FMD following 52 days of bed rest as well as a 17% decrease in femoral artery diameter in the control group only. Platts et al. [25] found an increase in popliteal artery %FMD of 6% following 49 days of bed rest, while Dyson et al. [38] observed no significant differences regarding %FMD following 56 days of bed rest. Nosova et al. [24] observed a 2% reduction in both popliteal artery and brachial artery %FMD following 5 days of bed rest. Hamburg et al. [13] found no significant differences regarding brachial artery %FMD following 5 days of bed rest, but did observe decreased reactive hyperemia in the forearm (1317 ± 404 to 1112 ± 260 mL/min) and the calf (28.5 ± 7.0 to 22.2 ± 8.7 mL/min/dL). Significant reductions in lower-body endothelium-dependent vasodilation assessed by blood flow changes in response to iontophoresis of an acetylcholine solution following bed rest were observed by Demiot et al. [38] (11.3%, 56 days in the control group only), Yuan et al. [49] (30%, 60 days), and Navasiolava et al. [42, 43] (< 50%, 7 days dry immersion).

#### Immobilization

In the five studies examining immobilization interventions, the interventions ranged from 8 days [44] to 4 weeks [45]. Four of the five studies examined lower-limb immobilization [37, 45–47], while one study examined forearm immobilization [44].

Birk et al. [35] found no significant differences in brachial artery baseline diameter or %FMD. Bleeker et al. [45] and Cohen et al. [37] found no significant differences regarding femoral artery %FMD after 4 weeks and 14 days of lower limb suspension, respectively. Rakobowchuk et al. [46] observed a significant increase in popliteal artery %FMD by 7.1% following 12 days of leg immobilization. Finally, Rytter et al. observed significantly reduced vascular conductance in the immobilized limb following 14 days of single leg immobilization.

## Discussion

Each of the five randomized controlled trials that were evaluated using the Cochrane Risk of Bias Tool were rated as having “low” risk of bias using the Cochrane RoB2 tool. For each of the other 14 studies evaluated using the NHLBI Study Quality Assessment Tools, one was rated as “good” [13] while 13 were reported as “fair” [20–22, 24, 25, 39–47]. Taken together, the results presented within this review can be interpreted with some confidence.

The results of the included studies show that reduction of daily steps reduced popliteal artery %FMD [21, 22], but that mixed results were shown for brachial artery %FMD [20, 21]. Further, detraining studies revealed decreases in microvascular function [39, 41], and no change in brachial artery %FMD [40]. In two out of four bed rest studies, lower limb %FMD was increased [25, 48], while one demonstrated a decrease in lower limb %FMD [24, 48]. One bed rest study also showed that brachial artery %FMD was reduced [23, 24], while one demonstrated no change [13]. Microvascular endothelial-dependent dilation was consistently reduced in all bed rest studies [38, 42, 43, 49]. Finally, two out of three immobilization studies demonstrated no difference in lower limb %FMD [37, 45] while one demonstrated an increase [46]. Limb immobilization resulted in no change in brachial artery %FMD [44] and a reduction in microvascular leg vasodilator responses [47].

Collectively, 13 of the above studies examined %FMD, and of those, four studies found decreases in %FMD following inactivity [20–22, 24]. No change in %FMD was observed by six of the studies [13, 23, 37, 40, 44, 45], while three studies reported increased %FMD following inactivity [25, 46, 48]. The mixed results of these studies likely highlight how the volume (duration and/or severity) of inactivity may impact %FMD. In an eloquent review by Booth et al. [50], the authors outline the physical inactivity spectrum from low-intensity physical inactivity to high-intensity physical inactivity. They note that high-intensity physical inactivity would be expected to show detrimental health effects within days, while low-intensity physical inactivity would take longer. We can apply this information from the Booth et al. [50] review when we compare the Boyle et al. [21] and Nosova et al. [24] papers. Both papers utilized a 5-day inactivity model; however, Boyle et al. [21] reduced steps to < 5000 per day while Nosova et al. [24] had subjects undergo bed rest. Bed rest, being a form of total or near-total inactivity, may stimulate greater vascular changes than step reduction alone. While both reduced lower limb %FMD following inactivity to a similar degree (3% and 2% reductions, respectively), only Nosova et al. [24] demonstrated a reduction in brachial artery %FMD. Similarly, when comparing Boyle et al. [21] with Bowden-Davies et al. [20], the more severe reduction in physical activity (Bowden-Davies et al. [20]) (this time in both duration and greater reduction in steps/day) resulted in decreases in both brachial and popliteal artery %FMD. This may also speak to differences in vascular adaptations to alterations in physical activity between upper and lower limbs. Indeed, at least with regard to exercise training, there does appear to be a disconnect between %FMD changes between upper and lower limb arteries [51, 52]. This may also be occurring with physical inactivity. Further, brachial artery %FMD does not correlate with lower limb (femoral and popliteal artery) %FMD [53]. Thus, while brachial artery %FMD is correlated with coronary endothelial function [54], it is not necessarily an indicator of systemic endothelial function [53]. It is pertinent to note that the brachial artery baseline diameter was reduced following 5 days of inactivity in Boyle et al. [21]. This reduced brachial artery baseline diameter may have compensated for the reduction in shear stress experienced following inactivity, as others have suggested [18, 21, 55, 56], thus resulting in no change in %FMD. However, while not %FMD, per se, these results may suggest that brachial artery vascular adaptations do occur in response to 5 days of reduced activity. Nonetheless, evidence in the literature demonstrates that as little as 5 days of reduced daily steps (< 5000 steps per day) decreases popliteal artery %FMD.

Vascular remodeling may occur with prolonged inactivity. A review by Thijssen et al. [18] demonstrates the chronic effects of physical activity to increase blood vessel diameter and chronic physical inactivity to decrease blood vessel diameter. Bleeker et al. [48], Platts et al. [25], and Rakobowchuk et al. [46] found increases in %FMD following 52 days of bed rest in the femoral artery, 49 days of bed rest in the popliteal artery, and after 12 days of single-leg immobilization in the popliteal artery, respectively. It is unclear why an increase in FMD was observed by Bleeker et al. [48] in this study but not by Dyson et al. [23], who carried out a 60-day bed rest study. However, Bleeker et al. [48] suggested that the increased FMD following 52 days of bed rest may have been due to increased nitric oxide sensitivity caused by the long duration of bed rest. Additionally, Bleeker et al. [48] observed a decrease in the diameter of the femoral artery following both 25 and 52 days of bed rest, which Dyson et al. [23] did not. This decrease in baseline diameter may account for the increase in %FMD, as %FMD is determined as the change in artery diameter from baseline to peak diameter post-ischemia [4]. Thus, blood vessels that have a smaller diameter tend to have a greater %FMD [4]. This same mechanism is proposed as the reason why no significant difference was seen regarding reduced daily steps in brachial artery %FMD by Boyle et al. [21], and may additionally explain the increase in %FMD observed by Platts et al. [25] and Rakobowchuk et al. [46].

Seven of the 20 studies examined microvascular function via laser Doppler flowmetry [38, 39, 41–43, 47, 49]. While limited in number, all of the included studies found significantly reduced microvascular function following inactivity. Peripheral microvascular function is associated with coronary vascular function [57]. Further, in patients with cardiovascular disease, coronary vascular function is independently linked to poor prognosis [58–61]. In a recent study, Young et al. [62] even found that peripheral microvascular function is negatively associated with having a major cardiovascular event in individuals with cardiovascular disease. Studies investigating the link between peripheral microvascular function on cardiovascular disease appear to be more prevalent in clinical populations [62–65]. Serne et al. [66] also demonstrated an inverse relationship between peripheral microvascular function and systolic blood pressure in healthy individuals. Elevated systolic blood pressure is linked to cardiovascular disease [67]. It is difficult to determine whether reductions in microvascular function due to acute inactivity are detrimental to vascular health in the long term. However, this information is useful in understanding how rapidly microvascular function is altered following inactivity, which may forecast chronic impairments in microvascular function if inactivity remains for a prolonged period of time.

It has been demonstrated that a reduction in shear stress leads to reduced endothelial function [68–70] and that shear stress plays an important role in maintaining a healthy endothelium [69–71]. Likewise, while markers of inflammation or oxidative stress were not outcomes of this literature review, previous research has shown that inactivity increases inflammation [72–74] and oxidative stress [16, 33, 73, 75]. Clapp et al. [76] demonstrated that inflammation, as measured by cytokine response, significantly decreased nitric oxide synthase bioavailability and antioxidant status, resulting in decreased endothelial function and increased oxidative stress [76]. Increases in oxidative stress are also known to decrease nitric oxide synthase bioavailability, further reducing endothelial function [77–79]. Thus, it is also possible that increased inflammation or oxidative stress is another mechanism by which short-term reductions in physical activity, detraining, or bed rest impact vascular health. This is an interesting area of research and should be explored further to delineate how these biomarkers may be impacting vascular function in response to inactivity.

While 14 of the 20 studies included utilized inactivity interventions of 30 days or less, 6 studies examined inactivity interventions lasting 52–60 days. Five of these studies employed bed rest interventions [23, 25, 38, 48, 49], while the remaining study utilized a detraining intervention [41]. Vascular remodeling has been demonstrated to occur after ~ 28 days of limb suspension [42], and thus the six studies that lasted 52–60 days in length may not necessarily be considered “short-term” interventions. However, we believe that these studies should still be included in this review for two reasons. First, to our knowledge, there have not yet been any other systematic reviews collectively examining the effects of inactivity interventions on endothelial function, regardless of timeline (short-term compared with longer-term). This indicates that these studies should be examined alongside shorter-term intervention studies to fully understand the time course of changes in endothelial function following inactivity. Second, while these six studies were longer than the other included studies, the directionality of significant differences remained consistent between studies regardless of length, with the exception of Bleeker et al. [48] and Platts et al. [25].

Overall, macrovascular and microvascular endothelial function demonstrated fairly consistent reductions following short-term physical inactivity. It is difficult to speculate whether these adaptations would remain with chronic inactivity and be indicative of a pathophysiological change versus a normal physiological response to acute reductions in activity. This is largely because no studies have assessed endothelial function both acutely and chronically in the same participants. Chronic diseases are generally slow to progress [80]. Thus, while chronic physical inactivity certainly leads to chronic diseases [50], acute inactivity likely only begins to initiate pathophysiological adaptations that may eventually develop into chronic diseases if inactivity is not reversed. While these changes in %FMD and microvascular function due to acute inactivity are small, they may still have clinical relevance and provide information on how short-term reductions in activity—such as occurs during acute hospital visits, vacations, or similar changes to daily routines that remove activity—may elicit dysfunctional consequences for those already at risk for CVD and atherosclerosis.

### Limitations

Several limitations are present in the current review. Firstly, 15 of the 20 studies were single-cohort pre-test post-test studies [13, 20–22, 24, 25, 39–47], allowing for a risk of bias due to lack of crossover or randomization. Additionally, all studies presented small sample sizes. Seven studies had 10 or fewer participants [24, 39–43, 45], ten studies had between 11 and 20 participants [13, 21, 22, 25, 38, 44, 46–49], and three studies had between 21 and 31 participants [20, 23, 37]. These limited sample sizes sum to a total of 273 participants for all 20 studies, an average of 14 participants per study. While we attempted to categorize studies into models, there were differences in severity and duration within models that may have contributed to differences in the magnitude of change in endothelial function. Of particular note, Bowden-Davies et al. [20] had a more severe reduction in daily steps (< 1500 steps/day) compared with the other two studies [21, 22] and demonstrated decreases in brachial artery %FMD, while Boyle et al.[21] only demonstrated decreases in popliteal, but not brachial, artery %FMD. Finally, owing to the limited number of studies available, different physical inactivity interventions, and the variety of measures of endothelial function employed, we were unable to complete a meta-analysis along with the systematic review.

### Future Directions

Future studies are required, examining the time course of changes in endothelial function following inactivity within all models of inactivity. Specifically, understanding how many hours of inactivity or the degree of inactivity via a meta-analysis would greatly advance this area of research. Additionally, future studies examining inactivity interventions within diseased populations at risk of endothelial dysfunction such as those with diabetes or cardiovascular disease are required. Diet and body weight were not considered in any of the studies. Reduction in physical activity may leave the subjects in an energy surplus, which may impair endothelial function. Studies demonstrate a link between increased body weight (or % body fat) and reduced endothelial function [81–83], which is likely being mediated via increased inflammation [84]. However, daily steps and dietary considerations did not appear to be well aligned in a large cohort study [85]; thus, not controlling for dietary intake during periods of inactivity may be more representative of a national population. Nonetheless, we cannot discount that, in the present systematic review, excess caloric intake may be impacting these results. Future studies should be more cognizant of controlling for the number of calories consumed. Many of the studies examined endothelial function in only one sex (men), which limits the applicability to women. Menopause is known to decrease endothelial function [86–88]; however, it is not clear how physical inactivity influences this impact. More studies are needed, examining the effects of physical inactivity on endothelial function in menopausal women.

## Conclusions

Acute physical inactivity interventions among adult populations may decrease measures of endothelial function such as brachial and popliteal artery FMD and microvascular function. While the speed at which these changes may occur for all individuals is not clear, significant decreases may occur as early as 5 days with reduction of physical activity. Additionally, those studies examining more severe, lengthier reductions of physical activity (e.g., bed rest rather than solely reduction of structured exercise), inconsistently demonstrated changes in endothelial function. More randomized controlled trials are needed to determine the length of time it takes for changes in endothelial function to be seen and the forms of physical inactivity that are most likely to cause these changes.

## Declarations

### Availability of Data and Materials

All studies from which data were examined are cited in the references section.
